# Personal resource gains: Effective coping builds academic buoyancy, and academic buoyancy builds achievement

**DOI:** 10.1111/bjep.70074

**Published:** 2026-03-10

**Authors:** David W. Putwain, Laura J. Nicholson

**Affiliations:** ^1^ School of Education Liverpool John Moores University Liverpool UK

**Keywords:** academic adversity, academic buoyancy, achievement, coping

## Abstract

**Background:**

Academic buoyancy is conceptualized as students' capacity to cope with academic challenges. Studies that examine how academic buoyancy and coping responses are reciprocally related, or that include relations with achievement, are lacking.

**Aims:**

The study examined reciprocal relations among academic buoyancy, coping and achievement.

**Sample:**

The sample comprised students aged 16–19 years in upper secondary education. At the first wave of data collection, there were 533 students (138 male, mean age = 16.4 years).

**Methods:**

Data were collected in five waves over two school years. Achievement was collected in waves one, three and five. Academic buoyancy and coping were measured in waves two and four via self‐report questionnaires.

**Results:**

Data were analysed in a structural equation model. Adaptive coping positively and non‐adaptive coping negatively predicted subsequent academic buoyancy. Additionally, academic buoyancy predicted subsequent achievement. A positive indirect relation from adaptive coping to subsequent achievement was mediated by adaptive coping.

**Conclusions:**

The findings show that personal resources can operate in cycles of resource gain as ‘co‐travellers’; effective coping can build academic buoyancy, and academic buoyancy can lead to better achievement. Moreover, study support for students could include ways to develop effective coping responses.

## INTRODUCTION

Formal and informal tests/exams are ubiquitous forms of student performance and progress assessment across many OECD countries (Mostafa, 2017). It is almost inevitable that many, if not all, students will find that a test/exam did not go as well as they had hoped at some point. The student could find the test/exam difficult, struggle to demonstrate their knowledge and skills, or not perform as well as expected. Students' responses to such challenges will determine subsequent educational flourishing or struggling (Skinner et al., 2013, 2016). The effective response to academic stress and challenge, such as an exam that did not go well, is conceptualized by Martin and Marsh (2008) as ‘academic buoyancy’, a proactive psychological asset that enables students to maintain their educational trajectory. While studies examining the coping strategies used in the face of challenging tests/exams are scarce (Putwain et al., 2024), they suggest that highly buoyant students employ more adaptive and fewer non‐adaptive strategies likely to overcome challenges and maintain a positive educational trajectory. Theoretically, greater use of adaptive and lesser use of non‐adaptive coping (i.e., ‘effective’ coping) would reinforce students' belief in their academic buoyancy, suggesting relations would be bidirectional. Moreover, academic buoyancy and effective coping could recursively benefit achievement, but studies that include achievement are scarce. In the present study, we address these limitations and examine bidirectional relations between academic buoyancy and coping alongside achievement.

### Academic buoyancy

As the definition above emphasizes, academic buoyancy is the capacity to overcome the challenges and stresses typical of education (Martin & Marsh, 2008, 2009). From a Conservation of Resource Theory (COR; Hobfoll et al., 2003, 2018) perspective, it can be characterized as a personal resource (alongside objects, conditions and energies) that leads to optimal human functioning. Consistent with this conceptualisation, Finnish Grade 6 students (pre‐transition to secondary school) who were highly buoyant showed lower school stress measured 6 months later, beyond that of temperament and prior achievement (Hirvonen et al., 2019). Moreover, highly buoyant Finnish Year 7 students (post‐transition to secondary school) showed lower school stress 18 months later in Grade 9, after controlling for parental education level and cognitive ability (Hoferichter et al., 2021). Studies of students using longitudinal samples of primary and secondary students from Australia, England and Finland show that buoyancy predicts achievement emotions (e.g., higher enjoyment and pride, lower anxiety and boredom) and study behaviours (e.g., planning, effort and persistence) that are conducive to learning and achievement (Hirvonen et al., 2020; Martin et al., 2010; Putwain et al., 2015, 2022). Several studies have shown that academic buoyancy is reciprocally related to cognate assets, including lower anxiety and greater competence beliefs, motivation and engagement (Bostwick et al., 2022; Martin et al., 2010, 2013; Putwain et al., 2016).

Academic buoyancy typically shows small (Putwain & Aveyard, 2018) or negligible (Collie et al., 2015) positive relations with achievement. As a proactive asset, academic buoyancy may play a role in protecting the achievement declines that would have been expected to result from adversity (Martin & Marsh, 2020; Putwain et al., 2020). The net result may be a small or negligible zero‐order correlation, but that need not imply that academic buoyancy is unrelated to achievement. Indeed, positive indirect relations between academic buoyancy and achievement, mediated by greater control (Collie et al., 2015), greater academic self‐concept (Colmar et al., 2019), and lower emotional exhaustion (Granziera et al., 2024), can be found even when zero‐order correlations are small or negligible. Studies using longitudinal designs to unpack directionality are scarce. In one such study, Putwain and Wood (2023) found that test scores positively predicted academic buoyancy, but academic buoyancy predicted achievement on only one of two waves.

### Academic coping

Coping broadly refers to executive regulation (i.e., conscious, deliberate and volitional) of one's cognition, emotion and behaviour, concerning a stressor (Compas et al., 2001; Skinner, 1999). A stressor is an event appraised as having personal relevance and which requires instrumental action by the person to ensure that goals and well‐being are not harmed or threatened (Folkman, 2008; Lazarus & Folkman, 1984). The academic stressor we focused on in the present study was a recent exam that had not gone as well as one had hoped; participants were instructed to report coping strategies used in response to this circumstance rather than coping strategies used more generally. Skinner et al. (2003) identified ≈400 coping strategies, many of which included overlapping categories and differing labels applied to similarly themed strategies (i.e., jingle‐jangle fallacies; Marsh et al., 2019). An alternative to looking at individual coping strategies is to combine them.

One approach differentiates between problem‐focused (attempts to deal with the stressor), emotion‐focused (regulating the emotions elicited by the stressful encounter) or avoidance strategies (Endler & Parker, 1990; Lazarus & Folkman, 1984). Problem‐focused coping positively correlates with achievement, but results are inconsistent for emotion‐focused and avoidance coping (Brdar et al., 2006; MacCann et al., 2011, 2012; Putwain et al., 2016). Another approach is to organize related strategies into higher‐order ‘families’ that are adaptive (effective at reducing distress and/or overcoming academic adversity) or non‐adaptive (maintaining or enhancing distress and/or academic adversity). Using this approach, Skinner and Saxton (2019) found that only the adaptive family of ‘problem‐solving’ was positively related to achievement. Non‐adaptive families were negatively related to persistence, self‐regulated learning and achievement.

We surmise two germane issues from the extant academic stress and achievement literature. First, studies that test for reciprocal relations between coping and achievement are lacking. From a COR framework, coping strategies would represent another facet of personal resources that would work alongside academic buoyancy in positive cycles of resource gain. Indeed, cognate resources, such as coping strategies and academic buoyancy, are likened to ‘co‐travellers’ (Hobfoll et al., 2018). Second, the nature of the coping and achievement relation is complex. Students typically use multiple coping responses when faced with a stressor, and it is not unusual for adaptive and non‐adaptive responses to be positively correlated (Thomas et al., 2017). For example, faced with a disappointing exam result, a student may use strategies to minimize distress *and* those designed to improve achievement. Inconsistent findings with achievement likely reflect the complex effects of some coping responses that reduce distress at the expense of effectively dealing with academic problems. Additionally, some strategies/families may be used centrally, while others are used peripherally. Rather than using single coping strategies/families, one potential solution is to consider the repertoire of adaptive and non‐adaptive coping responses (Cheng et al., 2014; Skinner et al., 2016).

### Academic buoyancy and coping

In Skinner and Saxton's (2019) transactional model, coping processes are triggered by the appraisal of stressful events (e.g., poor exam performance) as taxing or exceeding one's resources. Putwain et al. (2024) propose that academic buoyancy is an early‐stage input into this appraisal process. Highly buoyant students would be more likely to use a repertoire of coping responses in which the balance of adaptive strategies outweighs the non‐adaptive. Few studies have researched the link between academic buoyancy and coping. Putwain et al. (2012, 2016) found weak relations (*r*s = −.13 to .08) between academic buoyancy and three coping strategies commonly used when preparing for exams (‘task‐orientation and preparation’, ‘seeking social support’ and ‘avoidance’) in a sample of secondary school students. A later study by Putwain et al. (2024), also with secondary students and measuring a broader repertoire of nine coping strategies via the Cognitive Emotion Regulation Questionnaire (CERQ; Garnefski et al., 2002), showed stronger relations with academic buoyancy (*r*s = −.54 to .52).

None of these studies, however, considered the balance of adaptive and non‐adaptive coping responses. As students use adaptive and non‐adaptive responses together (Thomas et al., 2017), studying coping strategies in isolation may not reflect their deployment during adverse academic events. Accordingly, we created families (higher‐order factors) of adaptive and non‐adaptive coping from individual strategies and modelled their relations concurrently alongside academic buoyancy. This approach can provide a clearer indication of a person's overall coping approach rather than individual coping strategy scores.

Moreover, studies examining directional relations between academic buoyancy and coping are lacking. As coping processes occur cyclically, academic buoyancy would not only trigger effective coping responses, but responses that effectively reduce distress and/or deal with academic problems would reinforce capacity in an individual's buoyancy. Although feedback loops between stress appraisals and personal resources on the one hand, and learning and achievement outcomes on the other, were not included in Skinner and Saxton's (2019) transactional model, such recursive processes are widely acknowledged in appraisals of achievement situations (Lazarus & Folkman, 1984; Pekrun, 2024) and consistent with Hobfoll et al.'s (2018) COR approach to direct and indirect cycles of resource gain (or preventing resource loss).

Cycles of resource gain, or prevention of resource loss, can be optimized directly or indirectly (Hobfoll et al., 2003, 2018). Direct gains are self‐reinforcing, whereby resources such as academic buoyancy, effective coping or achievement build discretely over time. Indirect gains are mutually reinforcing, whereby resources in one area (e.g., academic buoyancy) contribute to the acquisition of resources (or minimizing resource depletion) in a second (e.g., coping or achievement). The COR conceptualisation of resource depletion or minimisation may be particularly relevant to the interpretation of zero‐order correlations between academic buoyancy and achievement; dips in achievement are minimized by buoyancy, which may be masked by small/negligible positive correlations.

**FIGURE 1:**
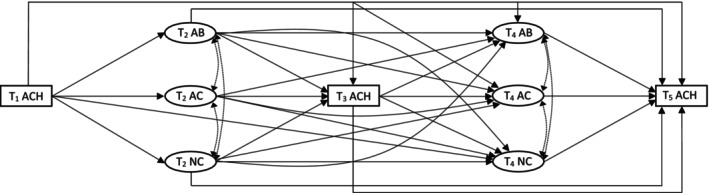
Paths tested in the SEM. SEM, structural equation modelling. ACH = achievement, AB = academic buoyancy, AC = adaptive coping, and NC= non‐adaptive coping.

By modelling novel reciprocal relations among academic buoyancy, adaptive and non‐adaptive coping, and achievement, we can establish if and how these personal resource ‘co‐travellers’ operate in indirect cycles of resource gain, or prevention of resource loss. Previous studies have examined cross‐sectional relations between academic buoyancy and coping (Putwain et al., 2012, 2016), and studies of reciprocal academic buoyancy and achievement are scarce (Putwain & Wood, 2023). We address these gaps in the literature using a ‘classic’ cross‐lagged panel model (CLPM) to assess bidirectional relations between academic buoyancy, coping and achievement, over a 12‐month interval, and in doing so, move beyond the use of individual coping strategies (see Figure 1). From a COR perspective, the autoregressive paths in Figure 1 represent direct, and the cross‐lagged paths represent indirect, resource gains (or prevention of resource depletion).

### Aims of the present study

Our study aimed to examine reciprocal relations among academic buoyancy, coping and achievement. Data were collected from a sample of upper secondary education students over two successive academic years in five waves (achievement at the first, third and fifth waves; academic buoyancy and coping at the second and fourth waves) separated by 6‐month intervals. We hypothesised that academic buoyancy would be positively and reciprocally related to adaptive coping (Hypothesis 1) and negatively and reciprocally related to non‐adaptive coping (Hypothesis 2). In addition, we hypothesised that academic buoyancy would show positive reciprocal relations with achievement (Hypothesis 3). As our higher‐order adaptive coping factor combines problem‐ and emotion‐focused approaches, which may dilute specific relations with achievement, we do not offer specific hypotheses here, but tentatively expect achievement to be positively related to adaptive and negatively related to non‐adaptive coping.

## METHOD

### Participants, sample and procedure

The sample comprised students in a tier of English upper secondary academic education (Years 12 and 13). Data were collected in five waves separated by 6 months. In the first wave (T_1_), students self‐reported their grades from high‐stakes secondary school exit exams taken at the end of Year 11 in two key subjects, namely English and mathematics. In the second wave (T_2_), approximately mid‐way through Year 12, students self‐reported academic buoyancy and coping. In the third wave (T_3_), achievement from end‐of‐year exams was collected from official school records. In the fourth wave (T_4_), approximately mid‐way through Year 13, students self‐reported academic buoyancy and coping. In the fifth wave (T_5_), achievement from standardized national upper‐secondary exit exams (typically used for university entrance) was collected from official school records. The project was approved by an institutional research ethics committee (17/EHC/001). School principals and individual participants provided written consent at T_2_ and T_4_. Participants could opt to participate in self‐report only or provide additional consent for us to collect achievement data.

At T_2_, there were 535 participants (mean age = 16.4 years, *SD* = .52); 533 reported achievement data from T_1_, and 439 provided permission to collect T_3_ achievement data. At T_4_, there were 450 participants (mean age = 17.4 years, *SD* = .50), comprising 318 returning participants from T_2_ and 132 new participants; 361 provided permission to collect T_5_ achievement data. Sociodemographic data for gender, ethnic heritage and free school meal eligibility (FSM: a proxy for low income) are shown in Table 1. In the years when data were collected, 34.5% (2021–2022) and 35.7% (2022–2023) of Year 12 and 13 students were from minority ethnic (i.e., non‐white) backgrounds, and 22.5% (2021–2022) and 23.8% (2022–2023), were eligible for FSM (Department for Education, 2022, 2023). Our sample was broadly representative of ethnic heritage compared to the national average and contained a smaller proportion of students from a low‐income background.

**TABLE 1:** Socio‐demographics of participants at all waves.

	Wave 1	Wave 2	Wave 3	Wave 4	Wave 5
Gender					
Male	138 (25.9%)	138 (25.8%)	109 (24.5%)	102 (22.6%)	85 (23.6%)
Female	374 (70.1%)	376 (70.2%)	315 (71.8%)	328 (73.1%)	258 (71.7%)
Prefer not to say	3 (.6%)	3 (.6%)	2 (.6%)	3 (.7%)	1 (.3%)
Non‐binary	18 (3.4%)	18 (3.4%)	13 (3.1%)	16 (3.6%)	16 (4.4%)
Ethnicity					
Black/British Black	80 (15%)	80 (15%)	70 (15.9%)	72 (16.0%)	49 (13.6%)
Chinese/British Chinese	3 (.6%)	3 (.6%)	2 (.5%)	3 (.7%)	3 (.8%)
South Asian/British South Asian	62 (11.6%)	62 (11.6%)	49 (11.2%)	50 (11.1%)	33 (9.2%)
British/European White	337 (63.2%)	339 (63.4%)	280 (63.8)	282 (62.7%)	242 (67.2%)
Other	27 (5.1%)	27 (5.0%)	19 (4.3%)	19 (4.2%)	13 (3.6%)
Mixed Heritage	24 (4.5%)	24 (4.4%)	19 (4.3%)	23 (5.3%)	20 (5.6%)
Free School Meals Eligibility	77 (14.4%)	77 (14.4%)	63 (14.4%)	60 (13.3%)	44 (12.2%)
Total	533	535	439	449	360

In total, 20% of the data were missing, and based on Little's test, *χ* ^2^(11) = 11.75 *p* = .962, they were assumed to be missing completely at random. Full Information Maximum Likelihood (FIML) was used in subsequent analyses to deal with missing data. Simulation studies have shown FIML to estimate unbiased model parameters under assumptions of MCAR (Jeličić et al., 2009; Nicholson et al., 2017). All data, materials and analysis code have been made publicly available at the Open Science Framework and can be accessed at: https://doi.org/10.17605/osf.io/xvfsy. The study was not preregistered.

### Measures

#### Academic buoyancy

The four‐item *Academic Buoyancy Scale* (ABS; Martin & Marsh, 2008) was used to measure the homonymous construct. Participants responded to four items, e.g., ‘I'm good at dealing with setbacks at college (e.g., bad mark, negative feedback on my work)’, on a five‐point scale (1 = ‘Strongly disagree’ to 5 = ‘Strongly agree’). Studies have shown strong evidence for the unidimensional factorial validity of this scale and good internal consistency (Granziera et al., 2022; Malmberg et al., 2013). In the present study, the internal consistency was also good (McDonald's *ω*s = .83 and 80 at T_2_ and T_4_, respectively).

#### Coping

The 36‐item CERQ (Garnefski et al., 2002) was used to measure coping. Emotion regulation used in response to a stressor represents coping strategies (Skinner & Zimmer‐Gembeck, 2007), and the CERQ was developed using coping strategies identified from the literature (Garnefski et al., 2001). The CERQ comprises 9 four‐item subscales, each relating to a specific coping strategy. Five coping strategies are considered adaptive (acceptance, positive refocusing, refocus on planning, positive reappraisal and putting into perspective) and four non‐adaptive (self‐blame, other‐blame, catastrophizing and rumination). Participants were instructed to report the extent to which they used those strategies after a recent exam that had not gone well. Participants responded to items (e.g., ‘I think about how to change the situation’ for refocus on planning) on a five‐point scale (1 = ‘Almost never’ to 5 = ‘Almost always’). Studies have demonstrated the factorial validity of the nine‐subscale structure, good internal consistency and predictive validity for psychological problems (Garnefski & Kraaij, 2018; Ireland et al., 2017). We combined CERQ subscales to create higher‐order adaptive and non‐adaptive coping factors. The internal consistency of these higher‐order factors, assessed using hierarchical McDonald's omega, was good (adaptive coping *ω*_H_s = .86 and .80, non‐adaptive coping = *ω*_H_s = .75 and .76, at T_2_ and T_4_, respectively).

#### Achievement

Students in English upper secondary education typically study three subjects (or their equivalent) and can choose from natural and social science, arts, humanities, and language subjects. As students can choose from multiple, sometimes unique, subject combinations, we aggregated T_3_ and T_5_ achievement across subjects to provide a mean grade. For T_1_ achievement, we chose English and mathematics grades as they are compulsory subjects until the end of Year 11 and have applicability to a wide range of subject combinations used in T_3_/T_5_ achievement. Participants self‐reported their T_1_ English and mathematics grades from national standardized secondary school exit exams (GCSE: General Certificate of Secondary Education). GCSE exams are graded from 1 to 9 (9 is the highest; 4 is considered a minimum pass). T_3_ achievement was from Year 12 school‐based end‐of‐year ‘mock’ exams used to generate a predicted grade for the offer of university study and summed to provide a mean score. T_5_ achievement used grades from national standardized upper‐secondary exit exams to confirm or disconfirm the offer of university study. T_3_/T_5_ achievement data were collected from official school records and converted to a seven‐point numeric scale (7 was the highest grade).^1^

#### Sociodemographic variables

Participants self‐reported gender, ethnic heritage, age and FSM eligibility.

### Analytic rationale

In preliminary analyses, longitudinal measurement invariance (i.e., equality of item‐factor loadings, item intercepts and item residual variances at T_2_ and T_4_) was tested for academic buoyancy and the nine CERQ subscales in a series of confirmatory factor analyses (CFAs). Equality of item‐factor loading and item intercepts is a prerequisite for modelling relations between variables over time (Widaman et al., 2010). Invariance of residual variances was shown for all variables (i.e., no substantial decline in model fit when equality constraints were added) apart from two CERQ subscales, where equality of intercepts were relaxed for two items. Invariance tests are reported in the Supporting Information (see Table S1).

A measurement model was then built comprising academic buoyancy, adaptive and non‐adaptive coping, and achievement alongside gender, age and FSM eligibility as demographic covariates. Gender was re‐coded as 0 = male, 1 = female, as there were insufficient responses to ‘other’ and ‘prefer not to say’ to model data. FSM was coded as 0 = not eligible and 1 = eligible. Academic buoyancy and coping were treated as latent variables, and all other variables as manifest. Academic buoyancy was modelled as a first‐order factor comprising the four ABS items. Adaptive and non‐adaptive coping were modelled as higher‐order factors. Adaptive coping was based on the CERQ subscales of acceptance, positive refocusing, refocus on planning, positive reappraisal and putting into perspective (each subscale comprising four items). Non‐adaptive coping was based on the four CERQ subscales of self‐blame, other‐blame, catastrophizing and rumination (also comprising four items per subscale).

Closely related coping strategies (e.g., positive reappraisal and putting into perspective; catastrophizing and rumination) can show low‐level cross‐loading to non‐target factors. In these circumstances, the cluster independence assumption of traditional CFA (i.e., that items can only load on the target factor and show zero loadings on non‐target factors) can be overly restrictive, suppress model fit and result in biased parameter estimates (Marsh et al., 2014; Morin et al., 2013). A plausible alternative is to use a set‐exploratory structural equation model (set‐ESEM). Set‐ESEM allows factors with a common set of indicators to be grouped (a ‘set’) and items within that set to cross‐load to non‐target factors. We used four sets to model higher‐order coping, namely the five adaptive coping strategies at T_2_ and T_4_ (sets one and two) and the four non‐adaptive coping strategies at T_2_ and T_4_ (sets three and four), with oblique target rotation and estimated using maximum likelihood.

A limitation of set‐ESEM, however, is that higher‐order models cannot be estimated. To transform the set‐ESEM solution into a higher‐order model, we used the CFA within ESEM (EwC) approach (Marsh et al., 2014, 2020). In the EwC model, unstandardised factor loadings from the set‐ESEM are used as starting values for lower‐order factors in a CFA, allowing for higher‐order factors to be estimated. Finally, based on the EwC model of higher‐order adaptive and non‐adaptive coping, a fully‐forward structural equation model was used to test the hypothesised relations in Figure 1. To establish whether possible cross‐lagged relations were an artefact of achievement, we tested an additional model, for comparative purposes, without achievement.

Model fit was established using the root mean square error of approximation (RMSEA), the standardized root mean residual (SRMR), the confirmatory fit index (CFI) and the Tucker–Lewis index (TLI). In a series of simulation studies, Hu and Bentler (1999) proposed RMSEA ≈ .06, SRMR ≈ .08, and CFI and TLI values ≈ .95 as indicating a good‐fitting model. These values should not be treated as definitive, especially with complex models based on naturalistic data (Lance et al., 2006). Model fit should be considered alongside other model parameters and theoretical considerations.

## RESULTS

Descriptive statistics are reported in Table 2. A measurement model of all study variables, alongside gender, FSM and age as possible socio‐demographic covariates, showed a reasonable fit to the data: $\chi_{\left(3247\right)}^{2}$ = 5356.11; RMSEA = .031, SRMR = .073, CFI = .924 and TLI = .913. Bivariate correlations are reported in Table 3. Academic buoyancy was positively correlated with adaptive and negatively correlated with non‐adaptive coping. Adaptive and non‐adaptive coping were negatively inter‐correlated. Female students reported lower academic buoyancy and adaptive coping, higher non‐adaptive coping, and higher T_1_ and T_3_ achievement. Older students reported higher adaptive coping (at T_4_ only).

**TABLE 2:** Descriptive statistics for academic buoyancy, adaptive coping, non‐adaptive coping and achievement.

Variable	Range	Mean	*SD*	Skewness	Kurtosis
T_2_ Academic Buoyancy	4–20	10.64	3.60	.33	−.48
T_2_ Adaptive Coping	20–100	54.39	11.89	.21	−.30
T_2_ Non‐adaptive Coping	16–80	40.91	10.64	.28	−.53
T_4_ Academic Buoyancy	4–20	10.77	3.70	.27	−.56
T_4_ Adaptive Coping	20–100	57.49	11.98	.17	−.46
T_4_ Non‐adaptive Coping	16–80	41.31	9.43	.12	−.73
T_1_ Achievement	1–9	6.56	1.08	.24	−.60
T_3_ Achievement	1–7	3.31	2.05	−.62	−1.06
T_5_ Achievement	1–7	3.38	1.11	−.13	−.38

**TABLE 3:** Bivariate correlations between academic buoyancy, adaptive and non‐adaptive coping, achievement, and demographics.

	1	2	3	4	5	6	7	8	9	10	11	12
1. T_2_ AB	—	.**70 (.05)**	**−.70 (.04)**	.**58 (.05)**	.**55 (.06)**	**−.44 (.05)**	−.01 (.05)	.12 (.06)	−.02 (.07)	**−.32 (.04)**	.01 (.05)	−.01 (.05)
2. T_2_ AC		—	**−.57 (.08)**	.**56 (.07)**	.**69 (.08)**	**−.43 (.08)**	.03 (.05)	.04 (.08)	−.04 (.08)	**−.24 (.06)**	.10 (.06)	.01 (.05)
3. T_2_ NC			—	**−.52 (.05)**	**−.56 (.07)**	.**69 (.04)**	−.03 (.06)	−.01 (.06)	−.02 (.07)	.**24 (.05)**	−.05 (.05)	.07 (.05)
4. T_4_ AB				—	.**81 (.05)**	**−.70 (.04)**	.08 (.07)	.03 (.09)	−.04 (.07)	**−.35 (.05)**	.06 (.06)	−.07 (.05)
5. T_4_ AC					—	**−.72 (.07)**	.08 (.07)	.03 (.09)	−.04 (.07)	**−.28 (.06)**	.**16 (.06)**	−.04 (.06)
6. T_4_ NC						—	.03 (.07)	.01 (.08)	−.08 (.06)	.**17 (.05)**	−.10 (.06)	−.01 (.05)
7. T_1_ ACH							—	.**78 (.08)**	.**48 (.06)**	.**05 (.02)**	.02 (.02)	−.03 (.01)
8. T_3_ ACH								—	.**82 (.08)**	.**09 (.03)**	−.01 (.04)	−.02 (.02)
9. T_5_ ACH									—	.05 (.02)	.02 (.03)	−.03 (.01)
10. Gender										—	.**11 (.04)**	.05 (.04)
11. Age											—	−.03 (.04)
12. FSM												—

*Note*: Standard errors in parentheses and coefficients in bold *p* < .05.

Abbreviations: AB, academic buoyancy; AC, adaptive coping; ACH, achievement; FSM, free school meals; NC, non‐adaptive coping.

The CLPM with achievement showed a reasonable fit to the data, $\chi_{\left(3253\right)}^{2}$ = 5068.46; RMSEA = .033, SRMR = .077, CFI = .921 and TLI = .917, and standardized regression coefficients are shown in Table 4. The CLPM without achievement showed a near identical model fit: $\chi_{\left(3025\right)}^{2}$ = 4720.12; RMSEA = .033, SRMR = .076, CFI = .922 and TLI = .917. Academic buoyancy was strongly correlated with adaptive and non‐adaptive coping (*r*s = .62 and .61 for adaptive, and −.66 and −.62 for non‐adaptive, coping at T_2_ and T_4_, respectively). Moreover, T_2_ adaptive coping positively (*β* = .25), and non‐adaptive coping negatively (*β* = −.19), predicted T_4_ academic buoyancy after accounting for the autoregressive relation with T_2_ academic buoyancy. T_2_ academic buoyancy, however, did not predict T_4_ adaptive or non‐adaptive coping. Female students showed higher achievement at T_1_ and T_3_ (*β*s = .12), lower academic buoyancy at T_2_ and T_4_ (*β*s = −.31 and −.14), lower T_2_ adaptive coping (*β* = −.23), and higher T_2_ non‐adaptive coping (*β* = .25). The same pattern of results was shown for the CLPM without achievement with only marginal differences (see Supporting Information, Table S2).

**TABLE 4:** Standardized coefficients for the cross‐lagged panel model with achievement.

	T_1_ ACH	T_2_ AB	T_2_ AC	T_2_ NC	T_3_ ACH	T_4_ AB	T_4_ AC	T_4_ NC	T_5_ ACH
T_1_ ACH		.06 (.03)	.03 (.04)	−.05 (.04)	.**49 (.04)**	.01 (.05)	.06 (.06)	.05 (.05)	.**21 (.05)**
T_2_ AB			. ***62 (.07)***	***−.66 (.05)***	.**16 (.07)**	.22 (.12)	−.06 (.13)	.18 (.12)	−.07 (.11)
T_2_ AC				***−.39 (.10)***	−.10 (.06)	.**25 (.10)**	.**61 (.11)**	−.10 (.10)	.01 (.11)
T_2_ NC					.02 (.05)	**−.19 (.09)**	−.18 (.11)	.**76 (.09)**	−.07 (.11)
T_3_ ACH						.08 (.04)	.03 (.04)	−.05 (.04)	.**46 (.05)**
T_4_ AB							. ***61 (.07)***	***−.62 (.07)***	.**23 (.09)**
T_4_ AC								***−.39 (.10)***	−.18 (.10)
T_4_ NC									.04 (.09)
Gender	.**12 (.04)**	**−.31 (.03)**	**−.23 (.05)**	.**25 (.05)**	.**12 (.04)**	**−.14 (.06)**	−.10 (.06)	.04 (.06)	.05 (.05)
Age	.04 (.04)	.04 (.05)	.**11 (.05)**	−.07 (.05)	−.03 (.04)	.05 (.05)	.05 (.06)	−.06 (.05)	.03 (.05)
FSM	−.07 (.04)	−.02 (.05)	−.02 (.05)	.07 (.05)	−.04 (.04)	−.10 (.05)	−.05 (.06)	−.02 (.05)	−.06 (.05)

*Note*: Standard errors in parentheses and coefficients in bold *p* < .05. Italicized coefficients in the CLPM are correlations.

Abbreviations: AB, academic buoyancy; AC, adaptive coping; ACH, achievement; FSM, free school meals; NC, non‐adaptive coping.

Academic buoyancy predicted subsequent achievement (T_2_ → T_3_, *β* = .16; T_4_ → T_5_, *β* = .23) beyond autoregressive relations with prior achievement. Relations from achievement to subsequent academic buoyancy were not statistically significant, albeit marginal (*p*s = .08). As a supplementary analysis, we checked for indirect relations from T_2_ coping to T_5_ achievement, mediated by T_4_ academic buoyancy. Indirect paths were statistically significant for adaptive coping, *β* = .058, *SE* = .033, 95% CIs [.003, 113], but not for maladaptive coping, *β* = −.043, *SE* = .027, 95% CIs [−.089, 002].

## DISCUSSION

The study aimed to examine relations between academic buoyancy and coping in the context of an exam that did not go well, alongside achievement. Previous studies have shown that academic buoyancy is positively related to adaptive and negatively to non‐adaptive coping strategies. We hypothesised that relations could be bidirectional. Not only would academic buoyancy trigger effective coping responses (i.e., greater adaptive and fewer non‐adaptive strategies), but effectively dealing with academic challenge would strengthen buoyant beliefs. Results partially supported Hypotheses 1 and 2. Adaptive coping positively, and non‐adaptive coping negatively, predicted subsequent academic buoyancy, but relations were not reciprocal; academic buoyancy did not predict subsequent adaptive or non‐adaptive coping. We also hypothesised bidirectional relations between academic buoyancy and achievement. Results partially supported Hypothesis 3. Academic buoyancy positively predicted subsequent achievement; relations from achievement to subsequent buoyancy were positive but marginally non‐significant. In addition, we found no statistically significant direct relations between coping and achievement. A positive indirect relation from adaptive coping to achievement was, however, mediated by academic buoyancy.

### Academic buoyancy and coping relations are not reciprocal

Although academic buoyancy is conceptualized as a student's capacity to cope effectively with educational challenges, few studies have examined the relation with coping responses (Putwain et al., 2012, 2016, 2024). Drawing on Skinner and Saxton's (2019) model of academic coping and COR theory (Hobfoll et al., 2003, 2018), we theorized bidirectional relations between academic buoyancy and coping. Results showed links from coping to buoyancy but not vice versa. Students who utilized more adaptive and less non‐adaptive coping following an exam that did not go as well as hoped for, viewed themselves as more academically buoyant 12 months later. This is an important finding as academically buoyant students are more likely to persist with challenging work (Granziera et al., 2022; Martin et al., 2010). From a COR perspective, students who employ effective coping skills (a skill‐based resource) come to believe they are better at dealing with educational challenges (i.e., academic buoyancy; a belief‐based resource). Like other appraisal models (Pekrun, 2024), Skinner and Saxton's (2019) model could be developed to acknowledge how the development of more or less effective coping strategies impacts subsequent appraisals and personal resources.

Academic buoyancy did not, however, relate to the use of effective coping 12 months later. One implication might be that academic buoyancy is not, within the context of Skinner and Saxton's (2019) model of academic coping, an early‐stage appraisal input. From a COR perspective, this finding might imply that belief‐based resources may not impact skill‐based resources in dealing with challenges. We reasoned that this was not the case. The principles behind academic buoyancy as an early‐stage appraisal input, or as an indirect route to resource acquisition, are sound. We speculate that the reason why we did not find expected links may lie in the 12‐month time interval. Coping responses to educational challenge unfold quickly (Duvenage et al., 2019). For instance, following an exam where a student found they could not answer many questions, they may initially blame themselves or their teacher. In the following days or weeks, however, a student may reappraise the situation and plan for what they need to do differently in the future. In these circumstances, a 12‐month interval between measurements of academic buoyancy and subsequent coping may be too long. If iterative cycles of reciprocal buoyancy and coping relations occur over shorter periods or fade over time, they will not be captured in the CLPM (see Dormann & Griffin, 2015).

### Buoyancy, coping and achievement

Previous studies have shown small/negligible relations between academic buoyancy and achievement that do not always reach statistical significance (Collie et al., 2015; Colmar et al., 2019) and longitudinal studies that can establish directionality are lacking (Putwain et al., 2022). In keeping with some previous studies (Putwain et al., 2015), we found academic buoyancy predicted subsequent achievement. From a COR theory perspective, this is another example of indirect resource acquisition, whereby personal resource beliefs (i.e., academic buoyancy) can positively impact educational flourishing. Academically buoyant students are more likely to invest more time and energy into behaviours aimed at overcoming educational challenges (e.g., effort, persistence), utilize more effective learning strategies (e.g., elaboration, retrieval practise) and experience emotions (e.g., enjoyment) known to assist achievement (Jansen et al., 2022). Achievement did not predict subsequent academic buoyancy, although results were in the expected direction and approached statistical significance. Such processes take time to enact, whereas the impact of achievement feedback on self‐perceptions is relatively immediate (Marsh et al., 2024). Just as relations from academic buoyancy to coping may weaken over 12 months, relations from achievement to buoyancy may weaken over a 6‐month interval.

In keeping with other studies (MacCann et al., 2011, 2012; Putwain et al., 2016), we also found small and non‐significant direct relations between coping and achievement. This is likely due to the presence of emotion‐focused strategies in our higher‐order adaptive (two of the five strategies) and non‐adaptive (all four strategies) coping factors. As the term implies, emotion‐focused strategies are methods of dealing (more or less effectively) with the emotions elicited by academic problems, but they do not deal with the impact itself. We suspect that problem‐focused strategies, with the potential to overcome educational challenges (e.g., planning, building competence/skills) individually or as a cluster, would likely show positive relations with achievement (Skinner & Saxton, 2019).

Adaptive coping did, however, show a positive indirect link to achievement, mediated by higher academic buoyancy. From a COR (Hobfoll et al., 2018) perspective, this indirect link reflects a cycle of personal resource gain whereby skill‐based resources (i.e., adaptive coping) foster belief‐based resources (i.e., academic buoyancy), which in turn support later achievement. This link between coping and achievement, operating through buoyancy, also supports our view that feedback loops are embedded in Skinner and Saxton's (2019) model of academic coping. In this model, appraisals, personal resources, coping and achievement outcomes are connected in ongoing, iterative and bidirectional cycles. The indirect link from adaptive coping to achievement also suggests that personal resources may contribute to achievement in parallel with coping processes, rather than operating solely through coping as implied in Skinner and Saxton's (2019) model.

### Limitations and recommendations for future studies

While our study added to the nascent literature around academic buoyancy and coping, there are nonetheless some limitations to highlight. First, we included nine coping strategies as measured by the CERQ. This was sufficient to capture a range of adaptive and non‐adaptive strategies (those focused on dealing with distress and those focused on dealing with the problem). Nonetheless, other relevant strategies may have been used by students but not included in the CERQ (e.g., behavioural coping strategies; Kraaij & Garnefski, 2019). Future studies could either utilize measures based on families of strategies to ensure broad coverage (Skinner et al., 2013) or select a range of adaptive and non‐adaptive strategies most relevant to the stressor or adversity under study.

Second, the 12‐month interval for the two waves of academic buoyancy and coping, and the 6‐month intervals with achievement, may not have been sufficiently sensitive to capture the cyclical nature of academic buoyancy, coping and achievement relations. Future studies could utilize more frequent assessments of academic buoyancy and coping processes (e.g., monthly or weekly) to establish an optimal time lag for modelling the processes that link them. In this respect, time‐intensive longitudinal survey designs modelling within‐person variance may be a useful complement. In addition, alternative modelling approaches that account for iterative cycles of resource gain (or minimizing resource depletion) within long measurement intervals, by using ‘contemporaneous’ paths (Marsh et al., 2024), could be considered.

Third, COR theory proposes that personal resources can either lead to resource gain or prevent resource loss (Hobfoll et al., 2003, 2018). One plausible reason why relations with achievement are often small/negligible is that academic buoyancy is protecting achievement against the declines that would have otherwise resulted from adversity. We were unable to empirically test the protective nature of buoyancy for achievement (and coping) as we did not collect data on the educational problem itself. Previous studies have used self‐report checklists of adversity (Martin & Marsh, 2020) and objective data for school absence and poor behaviour (Putwain et al., 2020) to establish the protective role of academic buoyancy. Future studies should adapt this approach and measure the specific adversity (or adversities) that academic buoyancy is theorized to assist coping with. This approach would enable a test of the extent to which academic buoyancy can, based on COR assumptions, prevent resource loss.

### Educational implications

Students who utilized effective coping (i.e., more adaptive and fewer maladaptive) responses developed a perception of themselves as buoyant and able to respond to educational challenges and stresses (like an exam not going well). To develop buoyant students (and the attendant benefits that would follow), the methods used by schools to educate and support students with their approaches to study could include training in academic buoyancy and coping with typical academic adversity. A *skills focus* could be used to teach students about effective coping strategies/families, and how and when to deploy them. For example, students could be shown how to deploy ‘keeping problems in perspective’ as a coping strategy (Braun et al., 2015). First, they are to generate a list of the different sorts of stressors, problems, adversities and challenges that adolescents face in all areas of life (both academic and non‐academic). Next, students order them from the most to the least severe. Finally, students reflect on the position of academic problems in the rank order compared to other problems.

Examples of persons students identify with (e.g., previous students from a similar background) who have learned to cope effectively would be used to support *vicarious* learning (Hernández et al., 2007). For example, audiovisual recordings of students from a similar socio‐demographic background to the target could be created. In these recordings, they describe the stressors, problems, adversities and challenges that they experienced, how they coped with these issues, and what they gained or learned from the situation. With several recordings, target students could be exposed to a range of different coping strategies and then asked to reflect on which coping strategies they could deploy in response to specific problems and adversities in the future. To develop competence, students should *practise* coping skills in less stressful or adverse conditions. For example, in advance of a class test, students could be asked to consider which coping strategies they have learned about (either through a skills focus or vicariously) that could be deployed before, during, and after the test, and when receiving feedback. To promote accurate judgement of buoyancy, students could *reflect* on which strategies were utilized, rate their effectiveness, and whether improvement is required (e.g., rehearsal, a deeper understanding of its practise is needed).

## CONCLUSION

Academic buoyancy was conceptualized as the capacity to cope with academic setbacks and challenges (Martin & Marsh, 2008). Our study found that students who used more adaptive and fewer non‐adaptive coping strategies to deal with an educational stressor (an exam that had not gone as well as hoped for) viewed themselves as being more academically buoyant 12 months later. In addition, academically buoyant students showed higher achievement. These findings support the COR theory of personal resources in one area contributing to resource acquisition in a second, cognate area (Hobfoll et al., 2003, 2018). In addition, frameworks such as Skinner and Saxton's (2019) model of academic coping could more explicitly incorporate recursive processes that connect coping and achievement back to students' stress appraisals and personal resources, while also accounting for the dynamic, time‐based nature of these interactions. Study support for students could use a skills focus and vicarious learning to develop a repertoire of effective coping responses.

## AUTHOR CONTRIBUTIONS

**David W. Putwain:** Conceptualization; investigation; writing – original draft; methodology; writing – review and editing; formal analysis; project administration; data curation. **Laura J. Nicholson:** Conceptualization; investigation; methodology; writing – review and editing.

## CONFLICT OF INTEREST STATEMENT

We have no known conflict of interest to disclose.

## Supporting information


Appendix S1.

## Data Availability

All data, materials and analysis code have been made publicly available at the Open Science Framework and can be accessed at: https://doi.org/10.17605/osf.io/xvfsy. The study was not preregistered.

## Footnotes

^1^
In Years 12 and 13, students study General Certificate of Education: Advanced Levels (A Levels) or Business and Technology Education Council (BTEC) qualifications alone or in combination. A Levels and BTECs are graded on a seven‐point letter scale.
