# Supplementary material for: Personal resource gains: Effective coping builds academic buoyancy, and academic buoyancy builds achievement
Source: Br J Educ Psychol. 2026 Mar 10;96(3):1206–22. doi: 10.1111/bjep.70074 (PMC13440989; doi:10.1111/bjep.70074)
Supplement: Supplementary file 1 [file BJEP-96-1206-s001.docx]

Table S1

*Invariance Tests for Academic Buoyancy and CERQ Coping Strategies*

|  | χ^2^(df) | RMSEA | SRMR | CFI | TLI | ΔRMSEA | ΔCFI | ΔTLI |
| --- | --- | --- | --- | --- | --- | --- | --- | --- |
|  |  |  |  |  |  |  |  |  |
| Academic Buoyancy^a^ | |  |  |  |  |  |  |  |
| Configural | 39.41 (13)*** | .055 | .025 | .982 | .962 |  |  |  |
| Metric | 41.94 (16)*** | .049 | .030 | .983 | .970 | -.006 | +.001 | +.008 |
| Scalar | 55.48 (20)*** | .052 | .040 | .976 | .967 | +.003 | -.007 | -.003 |
| Error | 71.67 (24)*** | .055 | .046 | .968 | .963 | +.002 | -.008 | -.004 |
|  |  |  |  |  |  |  |  |  |
| Positive Refocusing | |  |  |  |  |  |  |  |
| Configural | 34.08 (15)** | .044 | .034 | .986 | .974 |  |  |  |
| Metric | 37.79 (18)** | .040 | .035 | .986 | .987 | -.004 | +.001 | +.013 |
| Scalar | 43.39 (22)** | .038 | .042 | .984 | .980 | -.002 | -.002 | -.007 |
| Error | 45.00 (26)* | .033 | .044 | .986 | .985 | -.005 | +.002 | +.005 |
|  |  |  |  |  |  |  |  |  |
| Refocus on Planning | |  |  |  |  |  |  |  |
| Configural | 27.06 (15)* | .035 | .027 | .986 | .974 |  |  |  |
| Metric | 20.62 (18)* | .030 | .028 | .988 | .981 | -.050 | +.002 | +.007 |
| Scalar | 44.87(22)** | .039 | .045 | .973 | .966 | +.009 | -.015 | -.015 |
| Partial Scalar^b^ | 38.63 (21)** | .035 | .037 | .979 | .972 | +.005 | -.009 | -.009 |
|  |  |  |  |  |  |  |  |  |
| Acceptance |  |  |  |  |  |  |  |  |
| Configural | 67.96 (15)*** | .073 | .059 | .935 | .880 |  |  |  |
| Metric | 67.78 (18)*** | .065 | .059 | .939 | .906 | -.008 | +.004 | +.026 |
| Scalar | 86.48 (22)*** | .066 | .042 | .921 | .900 | +.001 | -.018 | -.006 |
| Partial Scalar^b^ | 75.51 (21)*** | .063 | .044 | .932 | .910 | -.002 | -.007 | +.004 |
|  |  |  |  |  |  |  |  |  |
| Rumination |  |  |  |  |  |  |  |  |
| Configural | 13.77 (15) | .000 | .025 | 1.000 | 1.000 |  |  |  |
| Metric | 16.45 (18) | .000 | .029 | 1.000 | 1.000 | .000 | .000 | .000 |
| Scalar | 22.55 (22) | .006 | .042 | .999 | .999 | +.006 | -.001 | -.001 |
| Error | 27.80 (26) | .010 | .039 | .989 | .989 | +.004 | -.001 | -.001 |
|  |  |  |  |  |  |  |  |  |
| Self Blame |  |  |  |  |  |  |  |  |
| Configural | 28.34 (15)* | .037 | .032 | .991 | .983 |  |  |  |
| Metric | 32.24 (18)* | .034 | .036 | .990 | .985 | -.003 | -.001 | +.002 |
| Scalar | 34.92 (22)* | .030 | .038 | .991 | .989 | -.004 | +.001 | +.004 |
| Error | 35.74 (26) | .024 | .041 | .993 | .993 | -.006 | +.002 | +.004 |
|  |  |  |  |  |  |  |  |  |
| Other Blame |  |  |  |  |  |  |  |  |
| Configural | 19.46 (15) | .021 | .038 | .990 | .981 |  |  |  |
| Metric | 25.08 (18) | .024 | .048 | .984 | .975 | +.003 | -.006 | -.006 |
| Scalar | 30.06 (22) | .023 | .049 | .982 | .977 | -.001 | -.002 | +.002 |
| Error | 29.95 (26) | .007 | .055 | .999 | .998 | -.016 | +.017 | +.011 |
|  |  |  |  |  |  |  |  |  |
| Catastrophising | |  |  |  |  |  |  |  |
| Configural | 37.48 (15)** | .047 | .032 | .978 | .960 |  |  |  |
| Metric | 42.77 (18)** | .045 | .040 | .976 | .963 | -.002 | -.002 | +.003 |
| Scalar | 47.90 (22)** | .042 | .041 | .975 | .968 | -.003 | -.001 | +.005 |
| Error | 50.16 (26)** | .037 | .042 | .977 | .975 | -.005 | +.002 | +.007 |
|  |  |  |  |  |  |  |  |  |
| *Note*. χ^2^ for all models *p* <.001. ^a^ Residual variances correlated between items 1 and 2 with similar wording at T_2_ and T_4_, ^b^ Constraint on item 2 relaxed | | | | | | | | |

Table S2

*Standardised Coefficients for the Cross-Lagged Panel Model Without Achievement*

|  | T_2_ AB | T_2_ AC | T_2_ NC | T_4_ AB | T_4_ AC | T_4_ NC |
| --- | --- | --- | --- | --- | --- | --- |
|  |  |  |  |  |  |  |
| T_2_ AB |  | ***.62 (.05)*** | ***-.66 (.04)*** | **.23 (.12)** | -.05 (.13) | .18 (.12) |
| T_2_ AC |  |  | ***-.43 (.07)*** | **.24 (.10)** | **.61 (.10)** | -.10 (.10) |
| T_2_ NC |  |  |  | **-.19 (.10)** | -.18 (.11) | **.76 (.09)** |
| T_4_ AB |  |  |  |  | ***.61 (.07)*** | ***-.62 (.06)*** |
| T_4_ AC |  |  |  |  |  | ***-.39 (.10)*** |
| T_4_ NC |  |  |  |  |  |  |
| Gender | **-.31 (.03)** | **-.23 (.05)** | **.25 (.05)** | **-.14 (.06)** | -.10 (.06) | .04 (.06) |
| Age | .04 (.05) | **.11 (.05)** | -.07 (.05) | **.05 (.05)** | .05 (.06) | -.06 (.05) |
| FSM | -.02 (.05) | -.02 (.05) | .07 (.05) | -.10 (.05) | -.05 (.06) | -.02 (.05) |
|  |  |  |  |  |  |  |
| *Note*. Standard errors in parentheses and coefficients in bold ***p* <.05**. Italicised coefficients in the CLPM are correlations. AB = academic buoyancy, AC = adaptive coping, NC = non-adaptive coping, and FSM = free school meals. | | | | | | |
